# Hydrological Controls on Ecosystem Dynamics in Lake Fryxell, Antarctica

**DOI:** 10.1371/journal.pone.0159038

**Published:** 2016-07-21

**Authors:** Radu Herbei, Alexander L. Rytel, W. Berry Lyons, Diane M. McKnight, Christopher Jaros, Michael N. Gooseff, John C. Priscu

**Affiliations:** 1 The Ohio State University, Columbus, OH, United States of America; 2 University of Colorado, Boulder, CO, United States of America; 3 Montana State University, Bozeman, MT, United States of America; University of Waikato (National Institute of Water and Atmospheric Research), NEW ZEALAND

## Abstract

The McMurdo Dry Valleys constitute the largest ice free area of Antarctica. The area is a polar desert with an annual precipitation of ∼ 3 cm water equivalent, but contains several lakes fed by glacial melt water streams that flow from four to twelve weeks of the year. Over the past ∼20 years, data have been collected on the lakes located in Taylor Valley, Antarctica as part of the McMurdo Dry Valley Long-Term Ecological Research program (MCM-LTER). This work aims to understand the impact of climate variations on the biological processes in all the ecosystem types within Taylor Valley, including the lakes. These lakes are stratified, closed-basin systems and are perennially covered with ice. Each lake contains a variety of planktonic and benthic algae that require nutrients for photosynthesis and growth. The work presented here focuses on Lake Fryxell, one of the three main lakes of Taylor Valley; it is fed by thirteen melt-water streams. We use a functional regression approach to link the physical, chemical, and biological processes within the stream-lake system to evaluate the input of water and nutrients on the biological processes in the lakes. The technique has been shown previously to provide important insights into these Antarctic lacustrine systems where data acquisition is not temporally coherent. We use data on primary production (PPR) and chlorophyll-A (CHL)from Lake Fryxell as well as discharge observations from two streams flowing into the lake. Our findings show an association between both PPR, CHL and stream input.

## Introduction

**Fig 1:**
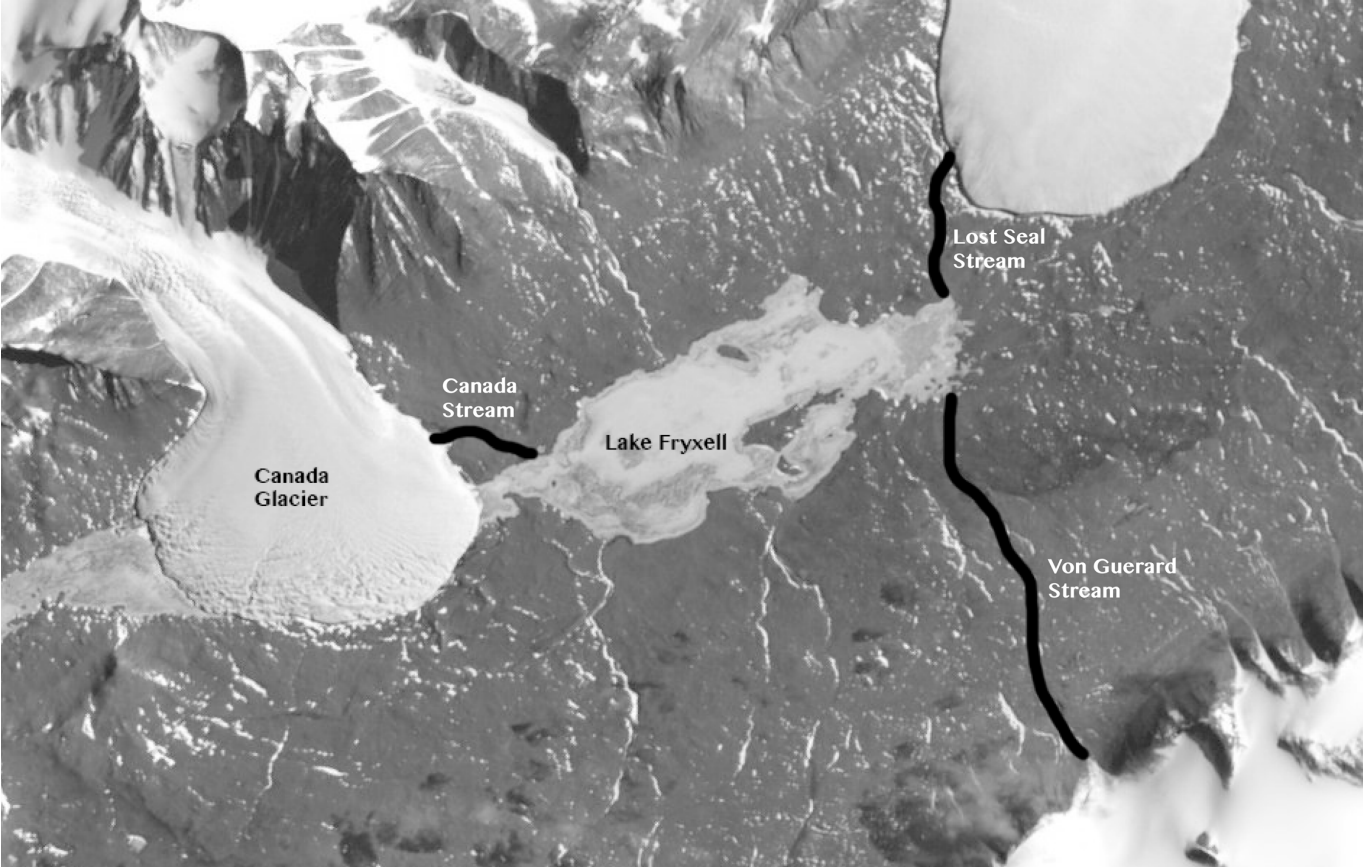
Aerial view of Taylor Valley, Antarctica and a schematic representation of Canada, Lost Seal and Von Guerard streams. Image available at http://earthobservatory.nasa.gov/IOTD/view.php?id=35535.

The relationship between physiochemical variations and ecological processes is one that has been of primary interest to aquatic ecologists. Changes in climatic variables such as temperature, precipitation and sediment can lead to changes in hydrological processes that, in turn, affect nutrient fluxes, light penetration and other important ecological parameters in aquatic systems. The significance of these physical drivers on changing ecological conditions can only be established if both physical processes and ecological response can be linked. This needed linkage is made even more difficult in extreme environments where year around measurements of biological parameters cannot be obtained. In this paper we advance our previous work [1] on understanding the impact of changing climate variables on the biology and chemistry of Lake Hoare, Antarctica, where biological/chemical variables were not measured at the same frequency as the physical ones. Taylor Valley is within the McMurdo Dry Valleys, which constitute the largest ice-free area in Antarctica [2]. The region has a mean annual temperature of −20°C and less than 3 cm of water-equivalent precipitation per year [3, 4]. Austral summer temperatures can, however, exceed 0°C for hours per day and for 4–12 weeks per year glacier melt flows through fixed stream channels to the closed-basin lakes: Lakes Bonney, Fryxell and Hoare (see Fig 1). We suggest a new modeling approach to the long-term ecological response of the lakes to changes in stream flow brought about by small, but significant changes in climate. We now focus on Lake Fryxell, another closed-basin lake in the Taylor Valley (∼ 78°S), Antarctica. The two lakes differ in size, age, biological production and routing of freshwater input [5].

Because the McMurdo Dry Valleys Long-Term Ecological Research (MCM-LTER, www.mcmlter.org) site program has been collecting meteorological, hydrological and limnological data since its inception in 1993, Taylor Valley is an excellent location to relate changes in climate to ecological variations in the lakes. Our previous work focused on Lake Hoare, the freshest of the Taylor Valley lakes and demonstrated that variations in photosynthetically active radiation (PAR) and dissolved reactive phosphate concentrations could be statistically related to changes in primary production (PPR) in the lake [1]. These findings strongly supported empirical evidence that in these lakes, that PPR is primarily driven by variations in PAR [6]. This is due in part to the very low light concentrations that exist in these perennially ice-covered lakes [7]. Declines in ice-cover transparency or increases in lake ice thickness reduce primary production rates, which in turn, limit the uptake of soluble nutrients, such as reactive phosphate.

The work presented here examines another of the Taylor Valley lakes, Lake Fryxell (see Fig 1). Unlike Lake Hoare, Lake Fryxell has a strong pycnocline and redoxocline (the lower portion of the lake is anoxic), is shallower, and is supplied with water by thirteen glacier melt-water streams from both the northern and the southern glaciers in the valley (see Fig 1). Of these streams, nine have been gauged since the late 1980s [8]. Annual average stream discharge is considered an important surrogate for climate variability, and is also thought to be important in driving in-lake processes [3, 8, 9]. However, long-term empirical evidence of this is currently lacking. Because one of the primary scientific goals of the MCMC-LTER program is to understand the linkage of climate variablity to ecological reponse, we applied similar ideas used in [1] that successfully related physio-chemical variables to ecological ones in Lake Hoare to evaluate the influence of stream discharge on the ecology of Lake Fryxell.

## Data

**Fig 2:**
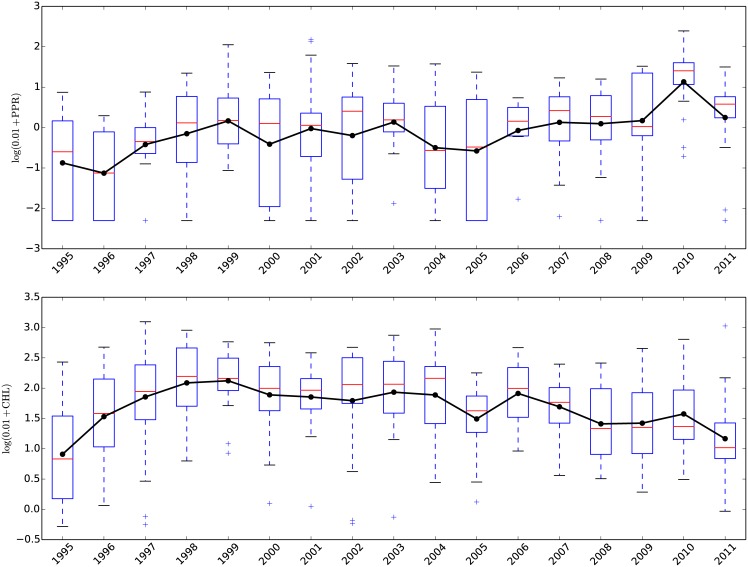
Boxplots for the observed primary production (PPR (*μgC*/(*L* × *day*)), top) and Chlorophyll A (CHL,(*μg*(Chlorophyll − A)/L) lower) values for the 1995–2011 period. The black line connects the average values for each season, marked with a black dot.

*Biological data.* We used the biological data collected by MCM-LTER researchers, available at www.mcmlter.org. Samples from Lake Fryxell were collected three to four times per flow season, in the austral summer, using standard limnological techniques (technical details regarding the data collection process can be found in the Limnological Methods for the McMurdo Long-Term Ecological Research Program compilation found at http://mcmlter.org/queries/lakes/lakeshome.jsp). We focused on two biological variables, primary production (PPR) and Chlorophyll A (CHL). For each of these, observations are available in a water column, roughly every 0.5m from just beneath the ice cover to the bottom of the lake. In our analysis we incorporate the observations above the chemocline, i.e., the boundary that defines the beginning of the anoxic zone as depth increases. The height of the chemocline was nearly unchanged through the time period of the study. Stream water enters the lake directly beneath the ice and because of this, only the top eleven meters of the water column in Lake Fryxell (above the chemocline) were considered when correlating these biological variables with stream discharge data. In Fig 2 we display annual box-plots for each variable. Our analysis takes into account observations taken during the months of October, November and December, for the 1995–2011 time period. This period was chosen, in part, because it represents the austral “spring” and “early summer” of the year, when nutrient input from the stream flow from the previous austral summer will have the most significance, i.e. prior to the beginning of the stream flow from the current season, when the biological measurements were made.

**Fig 3:**
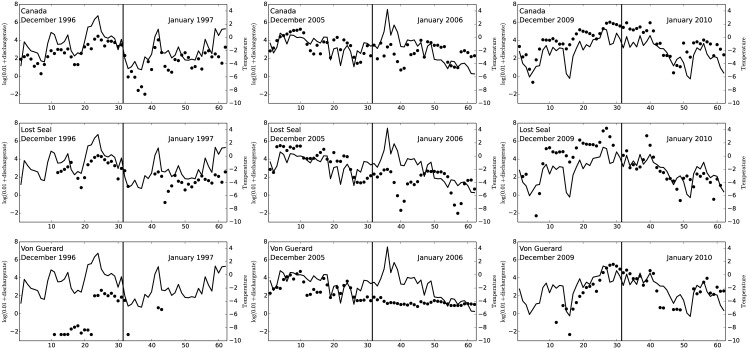
Log-Daily average discharge rates (black dots) for the Canada stream (top panels), Lost Seal stream (middle panels) and Von Guerard (lower panels) for the 1996–1997, 2005–2006 and 2009–2010 seasons. On each panel, we overlay the corresponding average daily temperature.

*Stream data*. As discussed above, there are thirteen streams flowing into Lake Fryxell, five of which supply most of the fresh water to the lake, most notably Aiken, Canada, Green, Lost Seal and Von Guerard [8]. There are gauges on nine of these streams (see www.mcmlter.org). The discharge data were collected every fifteen minutes from all of these gaged streams for the duration of the warm season each year of the research program (with frequent data gaps from a few hours to a few weeks in length). By temperate standards, these are not high volume streams, but we chose data from three of the largest volume streams, Canada, Lost Seal and Von Guerard, to analyze in this study (see Fig 1). Between 1995–2011, Canada, Lost Seal and Von Guerard streams generated between 12.4–29.3%, 13.5–29.6% and 0–13.6% respectively, of the total gaged flow into Lake Fryxell. During this period of record, the mean contribution from these three streams was 47.4%. In Fig 3 we show the discharge data (black dots) for three selected seasons, 1996–1997, 2005–2006 and 2009–2010. Each dot represents a daily average discharge, on a log-scale. The remaining seasons show a similar pattern, in general, with higher discharge rate values during the end of December, beginning of January period. As noted above and illustrated in Fig 3 there are significant periods where no data are available. We will discuss this issue further in the next Section.

**Fig 4:**
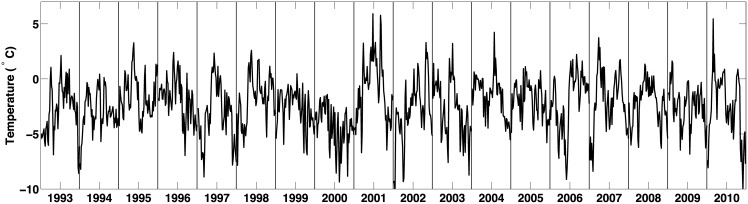
Time series of the daily averages of the air temperature at Lake Fryxell during the 1993–2010 period. For each year, we consider the months of December and January, which are used in our analysis.

*Temperature data.* In addition to the observations described above, we also use daily averages of the atmospheric temperature, collected near Lake Fryxell. The time series of these data are shown in Fig 4 and we explain in the next section how we incorporate them into our statistical model.

## Methods

### A functional regression approach

We begin by briefly describing the functional regression approach we pursue for this work. Abundant details and a comprehensive overview of the literature can be found in [10]. Consider a scalar response *Y* and assume that we have independent observations **Y** = (*Y*_1_,*Y*_2_,…,*Y*_*n*_). Departing from a classical linear regression approach, we consider several functional covariates *X*_*j*_(*t*), where *t* ∈ [0, *T*] and *j* = 1, 2,…,*J*. This is a common setting when analyzing longitudinal data in medicine, finance, biology, earth sciences, etc. The functional regression model takes the form

$$Y_{i}=\alpha +\sum_{j=1}^{J}\int_{0}^{T}\beta_{j}\left(t\right)X_{ij}\left(t\right)\;dt+\epsilon_{i}\;i=1,2,\cdots ,n\;,$$ (1)

where *α* is a scalar intercept and *β*_*j*_(*t*), *t* ∈ [0, *T*] is the functional regression coefficient corresponding to the j^th^ covariate *X*_*j*_(⋅), where *j* = 1, 2,…,*J*. In this paper we are concerned with the estimation of the functional coefficients *α* and *β*_*j*_(⋅). When applied to the available data from Lake Fryxell, the role of **Y** will be played by a biological variable (i.e., PPR, CHL) while the covariates *X*_*i*_(⋅) considered will be the discharge rates for the various streams that flow into Lake Fryxell.

Since functional spaces are infinite dimensional, estimation of the regression parameters *β*_*j*_(⋅) is typically done via a dimension reduction approach. The reason behind this is that one can always find functions *β*_*j*_(⋅) which will fit the model (1) perfectly, i.e., with zero residuals. Dimension reduction is done by projecting the coefficients *β*_*j*_(⋅) onto a finite dimensional space (ideally of dimension lower than the number of observations *n*). To that end, we assume the following basis function approximation

$$\beta_{j}\left(t\right)\approx \sum_{k=1}^{K}\gamma_{jk}\phi_{k}\left(t\right)\;,$$ (2)

where the functions {*ϕ*_*k*_(*t*), *t* ∈ [0, *T*], *k* ≥ 1} form an orthonormal basis, for the class of functions under consideration. Under the assumption that *β*_*j*_(⋅) for *j* = 1,…,*J* are square integrable functions, there are several popular choices for the basis {*ϕ*_*k*_(⋅), *k* ≥ 1}, such as wavelets, B-splines, Fourier, etc. Substituting Eq (2) into Eq (1) we get

$$Y_{i}=\alpha +\sum_{j=1}^{J}\sum_{k=1}^{K}\gamma_{jk}\int_{0}^{T}\phi_{k}\left(t\right)X_{ij}\left(t\right)\;dt+\epsilon_{i}\;i=1,\cdots ,n$$ (3)

Estimation of the regression parameters *β*(⋅) reduces now to estimating the basis expansion coefficients {*γ*_*jk*_, *j* = 1,…,*J*;*k* = 1,…,*K*}. Note that Eq (3) is a standard multivariate linear model, and can be fitted using various standard techniques. When the number of parameters, *J* × *K* is larger than the number of observations *n*, a regularization approach is suitable, see Chapter 5 of [11].

### Application and Results

A straightforward approach to fitting the model (3) requires apriori evaluation of the integrals

$$\int_{0}^{T}\phi_{k}\left(t\right)X_{ij}\left(t\right)\;dt$$

for each combination of indices *i*,*j*,*k*. These integrals can be approximated numerically over a fine equidistant time grid $T=\{0=t_{0}<t_{1}<t_{2},\ldots ,<t_{M}=T\}$ via

$$\int_{0}^{T}\phi_{k}\left(t\right)X_{ij}\left(t\right)\;dt\approx \sum_{m=0}^{M-1}\phi_{k}\left(t_{m}\right)X_{ij}\left(t_{m}\right)\Delta t$$

where Δt = *t*_1_−*t*_0_. Since the basis functions *ϕ*_*k*_(⋅) are known, this evaluation requires knowledge of the process *X*_*ij*_(⋅) at all time points {*t*_*m*_}. In our application, the functional covariates *X*_*ij*_(⋅) will be the discharge rates from Canada, Lost Seal and Von Guerard streams. Upon inspection of the discharge rate observations for Lake Fryxell, we note that there are some time intervals where discharge was not observed. Our methodology will account for this as we explain below.

In a preliminary step, we estimate the discharge profiles *X*_*ij*_(*t*_*m*_) for each season *i* and stream *j* at all time points *t*_*m*_. It has been established that discharge rates in Taylor Valley are highly correlated to air temperature [12]. In Fig 4 we display a time series for the daily average air temperature observed at the Lake Fryxell meteorological station during the months of December and January, from 1994 until 2010. The strong association between temperature and discharge rates is reflected in Fig 3 where we overlay a time series of the temperature observations and log-discharge rates for three summers (Dec. 1996–Jan. 1997, Dec. 2005–Jan. 2006 and Dec. 2009–Jan. 2010). Although not displayed, a very similar pattern between stream discharge and air temperature is present throughout the entire study period.

The discharge data set is plagued by many missing observations. In our approach to understand the uncertainty in the biological variables within Lake Fryxell we do require a *complete* record of discharge observations for every season, from 1994 until 2010. We can use the previously discussed association between temperature and discharge to predict the values of the missing variable at the times *t*_*ij*_ as required by model (3). We suggest the following statistical model. We fix a constant *ζ* > 0 and denot DR = log(ζ + Discharge), in order to avoid taking logarithms of observed discharge rates which were equal to zero. We use a Gaussian Markov Random Field (GMRF) specification for the underlying discharge rate process as follows,

$$\begin{array}{c} \left\{\begin{array}{ccc} {\text{DR}}^{\text{obs}}\left(t^{\left(l\right)}\right) & = & \text{DR}\left(t^{\left(l\right)}\right)+\xi \left(t^{\left(l\right)}\right)\;l=1,2\ldots ,L\\ \text{DR}(\cdot ) & ∼ & \text{GMRF}(\text{mean}=\delta_{0}+\delta_{1}T\left(\cdot \right),\;\text{covariance}=\Sigma )\;. \end{array}\right. \end{array}$$ (4)

**Fig 5:**
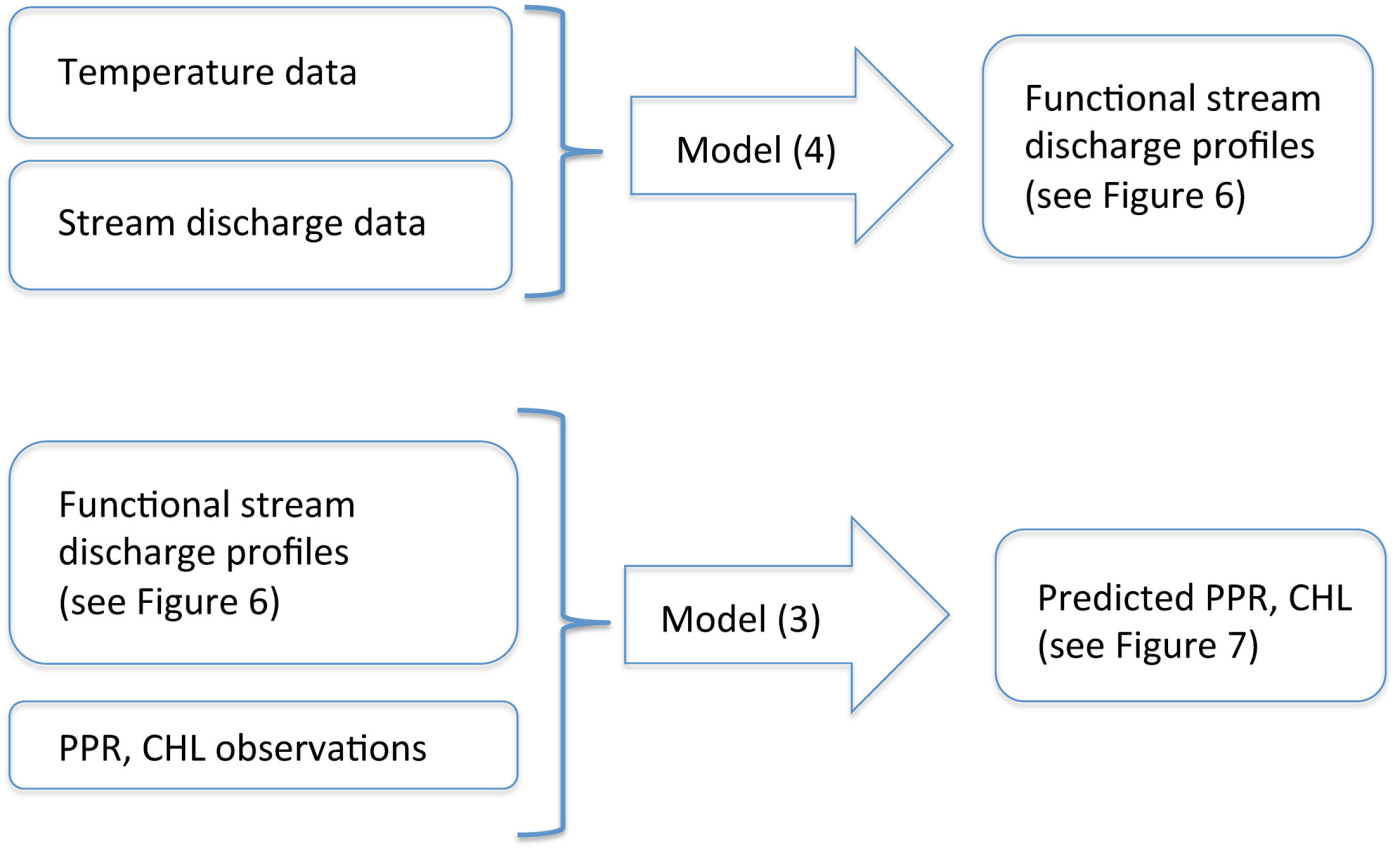
A schematic representation of the functional regression approach described in Section.

We fit this model for each austral summer, from 1994 until 2010, independently. In each season, in the model (4) above, DR*^obs^*(*t*^(*l*)^)represents a discharge observation at a time point $t^{\left(l\right)}\in T$, and *L* represents the total number of discharge observations for the summer under study. We use $\text{DR}\left(\cdot \right)=\{\text{DR}\left(t_{m}\right),\;t_{m}\in T\}$ to denote the unknown underlying discharge rate process and $T\left(\cdot \right)=\{T\left(t_{m}\right),\;t_{m}\in T\}$ represents the temperature process. The errors *ξ*(⋅) are assumed to be independent and identically distributed, Gaussian variates with mean zero and unknown variance $\sigma_{e}^{2}$. GMRF models are a popular choice when specifying prior distribution models for random fields, in part due to their flexibility and computational efficiency [13]. The covariance matrix *Σ* is specified via its inverse, the precision matrix *Σ*^−1^, which is sparse. The role of DR^obs^(·)is played by observations collected from the Canada, Lost Seal and Von Guerard streams. In each case, model (4) is fitted in a Bayesian framework. This approach is schematically described in Fig 5.

**Fig 6:**
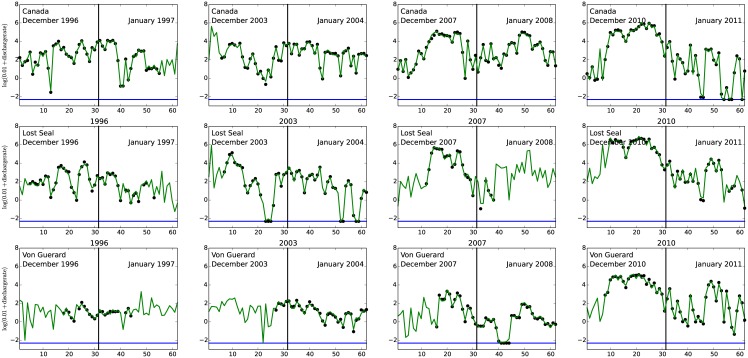
Observed log-discharge rates (dots) for the Canada stream (top panels), Lost Seal stream (middle panels) and Von Guerard stream (lower panels), for the 1996–1997, 2003–2004, 2007–2008 and 2010–2011 seasons. The green curves represent the predicted log-discharge for the four selected seasons. The horizontal blue line is at log(0.01) which represents zero discharge.

In Fig 6 we display the observations DR^obs^(·)(black dots, on a log-scale) as well as the corresponding fitted processes DR(·) (green curves), for the Canada, Lost Seal and Von Guerard streams, for four different austral summers months (December and January).

**Fig 7:**
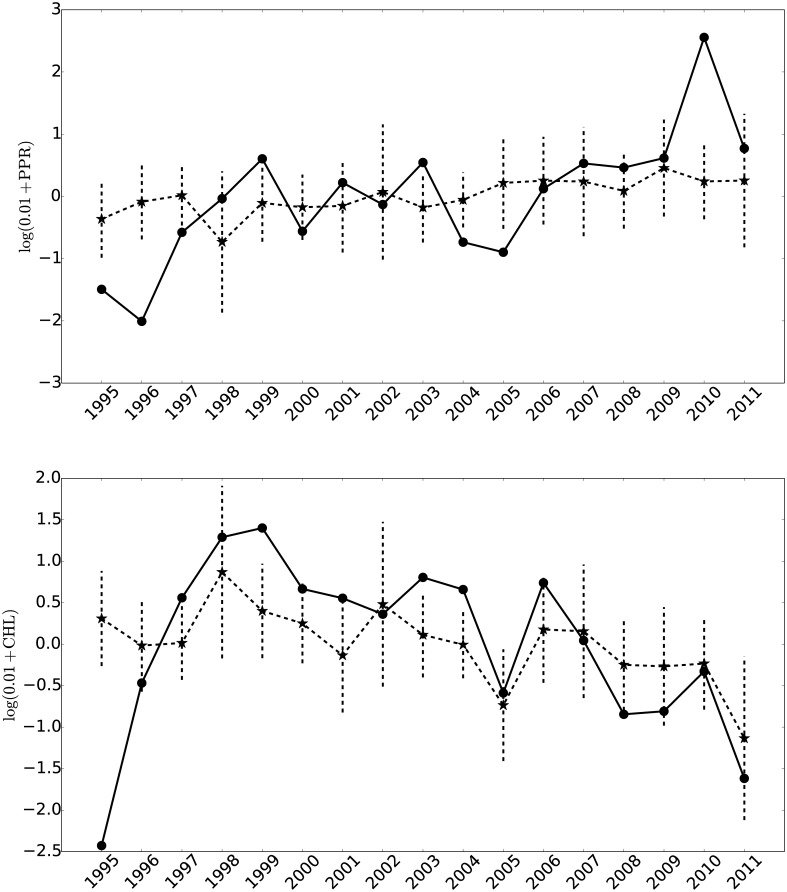
Observed (dots) and fitted (stars) PPR (top panel) and CHL (lower panel) values for the 1995–2011 period. The vertical dashed lines represent predicted value ± 2 × standard errors for each season.

Once we have obtained estimates for the stream discharge profiles for both the three streams under consideration, at all time points *t*_*m*_, we used these as functional covariates in the regression model described above. The response variable **Y** is the seasonal average (October-December) for the two biological variables under study, PPR and CHL. Given that the amount of data is very limited (just over a decade), for each response variate, we fit three models, one for each of the streams under consideration: Canada, Lost Seal and Von Guerard. In each case we used a one year lag between the stream data and the biological variate. This was done, as we noted previously, because the biology of these lakes in the early part of the austral summer is probably driven by nutrient input from the previous season [9], as we discuss below. In Fig 7 we show the observed values for the biological variables PPR and CHL as well as the corresponding predicted values for the period 1995–2011. These are obtained by averaging the predicted values for the three models, corresponding to the Canada, Lost Seal and Von Guerard streams. The vertical dashed lines correspond to predicted value ± 2 × standard errors for each season. The model fit is assessed via the coefficient of determination. We estimate the average (over the three models) R-squared to be 29.45% (for CHL) and 13.17% (for PPR), indicating that stream discharge plays a significant role in the primary production of Lake Fryxell.

## Discussion

In this manuscript we present a statistical approach to modeling the association between stream discharge and the biological production in lake Fryxell, Antarctica. Our approach is based on a functional regression model, which requires complete (functional) observations of stream discharge over the period of interest (December-January of each austral summer, in this case). Although we have incomplete discharge observations, we are able to obtain functional estimates of the stream discharge (see Fig 6) via a separate statistical model. Both models used in this manuscript, described via Eqs (3) and (4) are generally applicable, see for example [1] for a similar approach. Our results support the hypothesis that stream discharge into Lake Fryxell provides an charge of nutrients which in turn supports primary production during the austral springs.

Annual variation of stream flow into a permanently ice-covered, closed-basin lake such as Lake Fryxell has a number of important ecological consequences. Increased stream flow introduces higher nutrient concentrations into the lake’s surface waters and it might also introduce high amounts of suspended matter into the lake as well. In the case of the former, this process should lead to potentially increased primary production in the surface portion of the euphotic zone [9]. Passed work has demonstrated that in some cases, increased flow may initially decrease both primary production and biomass concentrations in the surface waters of the lake, due to a decrease in PAR from the introduction of the suspended materials [9]. PAR is the most important factor influencing primary production in the McMurdo Dry Valleys [1, 6] but the lakes also have nutrient deficiencies [14]. Increased stream flows are usually associated with warmer austral summer temperatures. These in turn are summers when the ice cover thins, thus affecting PAR [15]. Thus, warmer austral summers can have two important positive impacts in the lake’s surface waters: increasing the nutrient input as well as decreasing ice thickness which increases light penetration into the lakes.

The three streams under consideration have mean dissolved inorganic nitrogen (DIN) and soluble reactive phosphate (SRP) as summarized in Table 1, see [16]. In contrast, the surface waters of Lake Fryxell have DIN = 0.05*μ*M, SRP = 0.08*μ*M [17]. Clearly, the annual stream input of nutrients, especially DIN, in the case of Lake Fryxell is very important to the overall biological production in the surface waters [14]. In Lake Fryxell, nutrients do diffuse upwards from the anoxic zone across the chemocline into the euphotic zone, but this flux is only significant in driving production in the deeper CHL−A maximum at or directly above the chemocline [14, 17]. It has been established that half of the lake’s primary production is supported by nutrient input from streams and glacier melt [16]. Clearly higher flow years will increase this nutrient input. Thus, unless the streams entering the lake contain higher amounts of suspended material, high stream flow years should enhance biological production in lakes such as Lake Fryxell. Although extensive studies have not been undertaken, the data that are available suggest that even during the highest stream flows, only a small number of streams in Taylor Valley have the ability to transport high amounts of particulates, and these drop out rapidly in the lakes [9, 18].

**Table 1:** Summary of DIN and SRP values for the Canada, Lost Seal and Von Guerard streams.

	DIN	SRP
Canada	0.99*μM*	0.29*μM*
Lost Seal	3.06*μM*	0.84*μM*
Von Guerard	1.96*μM*	1.39*μM*

The significance of the year lag can be explained as follows. In the austral spring (October―November), after the sun rises, primary production begins. However, because glacier melt, and hence stream flow does not occur generally until late November, and the maximum flows until mid-late December into early January, the yearly pulse of primary production must be driven by nutrient input from the previous flow season, and, in some lakes via upward diffusion of nutrients [14]. Thus, prior to the austral summer’s infusion of new water, the lakes operate ecologically as a closed-system, without any external nutrient input. Therefore, increased flow and increased nutrient loading sets the stage for an increase in primary production in the following austral spring. Foreman et al. [9] referred to this process as a “recharge” event, whereas high flow events play an important role in supplying the lake ecosystems with pulses of nutrients. Discerning the impact of these “pulse” events on ecosystem structure and function is a major theme of the MCM-LTER (www.mcmlter.org). As the climate continues to warm, it is anticipated that these pulse events will become more frequent, thereby increasing the connectivity of the various landscape units through increased cryospheric melting/thawing [19]. This modeling effort suggests that an increase in pulse frequency may lead to increased biological production in the lakes.

## Supporting Information

S1 Data The data sets used in this analysis are available in the file S1_Data.zip. We provide the temperature observations, the PPR and CHL observations, as well as the discharge rate data from the Canada, Lost Seal and Von Guerard streams.
(ZIP)
